# Spinal Manipulative Therapy Alters Brain Activity in Patients With Chronic Low Back Pain: A Longitudinal Brain fMRI Study

**DOI:** 10.3389/fnint.2020.534595

**Published:** 2020-11-19

**Authors:** Wenli Tan, Wei Wang, Yuchan Yang, Yilei Chen, Yingjie Kang, Yanwen Huang, Zhigang Gong, Songhua Zhan, Zeng Ke, Jianwei Wang, Weian Yuan, Weiyuan Huang, Chishing Zee, Zikuan Chen, Bihong T. Chen

**Affiliations:** ^1^Department of Radiology, Shuguang Hospital Affiliated to Shanghai University of Traditional Chinese Medicine, Shanghai, China; ^2^Department of Tuina, Shuguang Hospital Affiliated to Shanghai University of Traditional Chinese Medicine, Shanghai, China; ^3^Institute of Traumatology and Orthopedics, Shuguang Hospital Affiliated to Shanghai University of Traditional Chinese Medicine, Shanghai, China; ^4^Department of Radiology, Hainan General Hospital, Haikou, China; ^5^Department of Radiology, Keck School of Medicine, University of Southern California, Los Angeles, CA, United States; ^6^Department of Diagnostic Radiology, City of Hope National Medical Center, Duarte, CA, United States

**Keywords:** functional magnetic resonance imaging (fMRI), chronic low back pain (CLBP), mechanical stimulus, spinal manipulative therapy, brain function, default mode network (DMN)

## Abstract

**Background:** Spinal manipulative therapy (SMT) helps to reduce chronic low back pain (cLBP). However, the underlying mechanism of pain relief and the neurological response to SMT remains unclear. We utilized brain functional magnetic resonance imaging (fMRI) upon the application of a real-time spot pressure mechanical stimulus to assess the effects of SMT on patients with cLBP.

**Methods:** Patients with cLBP (Group 1, *n* = 14) and age-matched healthy controls without cLBP (Group 2, *n* = 20) were prospectively enrolled. Brain fMRI was performed for Group 1 at three time points: before SMT (TP1), after the first SMT session (TP2), and after the sixth SMT session (TP3). The healthy controls (Group 2) did not receive SMT and underwent only one fMRI scan. During fMRI scanning, a real-time spot pressure mechanical stimulus was applied to the low back area of all participants. Participants in Group 1 completed clinical questionnaires assessing pain and quality of life using a visual analog scale (VAS) and the Chinese Short Form Oswestry Disability Index (C-SFODI), respectively.

**Results:** Before SMT (TP1), there were no significant differences in brain activity between Group 1 and Group 2. After the first SMT session (TP2), Group 1 showed significantly greater brain activity in the right parahippocampal gyrus, right dorsolateral prefrontal cortex, and left precuneus compared to Group 2 (*P* < 0.05). After the sixth SMT session (TP3), Group 1 showed significantly greater brain activity in the posterior cingulate gyrus and right inferior frontal gyrus compared to Group 2 (*P* < 0.05). After both the first and sixth SMT sessions (TP2 and TP3), Group 1 had significantly lower VAS pain scores and C-SFODI scores than at TP1 (*P* < 0.001).

**Conclusion:** We observed alterations in brain activity in regions of the default mode network in patients with cLBP after SMT. These findings suggest the potential utility of the default mode network as a neuroimaging biomarker for pain management in patients with cLBP.

**Clinical Trial Registration:** Chinese Clinical Trial Registry, identifier ChiCTR1800015620.

## Introduction

Chronic low back pain (cLBP) is one of the most common ailments, affecting ~13 in 100 people at some point in their lives, and contributes to great socioeconomic costs around the world (Shmagel et al., 2016). The prevalence of cLBP increases significantly with age, from 4.2% between the ages of 24 and 39 to 19.6% between the ages of 20 and 59 (Meucci et al., 2015). Most patients with cLBP undergo conservative treatment for pain relief whenever feasible (Rainville et al., 2009; Xu et al., 2013). Spinal manipulative therapy (SMT), one of several complementary non-surgical treatment methods for cLBP (Bervoets et al., 2015; Lee et al., 2017), is performed by trained practitioners who apply a controlled force to the spine using their hands or a specialized device for pain relief. SMT not only helps to reduce pain but also improves the physical functioning of patients with cLBP (Bronfort et al., 2010; Kong et al., 2012). Ruddock et al. (2016) reported that SMT was more effective at relieving low back pain than a sham intervention. However, the underlying mechanism of pain relief and the neurological response to SMT remains unclear.

Brain functional magnetic resonance imaging (fMRI) has been used to study the potential alterations in brain function in patients with cLBP (Yu et al., 2014; Kregel et al., 2015; Konno and Sekiguchi, 2018; Ng et al., 2018). For example, a study by Giesecke et al. (2004) demonstrated that patients with cLBP had more extensive neuronal activation in pain-related brain cortical areas than healthy controls in response to the application of pressure with a thumbnail. Another study by Sharma et al. (2011) used a straight leg raise maneuver to stimulate pain during fMRI and detected increased brain activity within the anterior cingulate gyrus, bilateral insular regions, right thalamus, basal ganglia, and sensorimotor regions. In addition, published literature has also provided evidence for increased brain activation in several brain regions after the application of mechanical stimuli to the low back area (Kobayashi et al., 2009; Manchikanti et al., 2009). One study by Ellingsen et al., which focused on the emotional changes in patients with cLBP, reported patients having less fear to move around after one SMT session (Ellingsen et al., 2018). Furthermore, our prior study found diminished activity in the prefrontal cortex and cerebellum in patients with lumbar disc herniation treated with SMT (Yuan et al., 2015). Therefore, the literature to date has shown functional alterations in the brain in patients with cLBP. However, few studies have focused on alterations in brain activity after SMT in patients with cLBP. Thus, there is limited information on how cLBP and SMT alter brain activity.

Mechanical pressure applied to the low back area is commonly used in clinical practice for assessing and treating low back pain. It is also used in pain research to simulate the back pressure experienced by patients (Kobayashi et al., 2009; Meier et al., 2014; Mansour et al., 2018; Ng et al., 2018). Here, we performed a prospective longitudinal study utilizing brain fMRI to assess alterations in brain activity in patients with cLBP after SMT, manually applying a mechanical spot pressure stimulus during fMRI scanning. We enrolled patients with cLBP and obtained assessments at three time points: prior to SMT, after one SMT session, and after six SMT sessions. Assessments at each time point for the patients with cLBP included a brain fMRI scan and two clinical questionnaires to assess pain and quality of life. We also enrolled age-matched healthy controls without cLBP and obtained one brain fMRI scan in a similar setting with real-time mechanical spot pressure applied.

## Materials and Methods

### Participants

This was a prospective longitudinal study utilizing fMRI to access brain responses to pain management using SMT in patients with cLBP. Participants were enrolled in this study from May 1, 2018, to December 31, 2018.

We enrolled patients with cLBP (Group 1) from our outpatient pain clinics. according to the following eligibility criteria: (1) right-handed; (2) 20–70 years of age; (3) visual analog scale (VAS) score ≥ 30/100; (4) Chinese Short Form Oswestry Disability Index Questionnaire (C-SFODI) score ≥ 20% (Zheng et al., 2002); (5) no pharmacotherapy, physical therapy, or SMT for pain 1 month prior to enrollment; (6) able to understand and sign the written informed consent; (7) clinical diagnosis of cLBP with pain and/or discomfort in their low back for ≥3 months (Burton et al., 2006). Exclusion criteria for the Group 1 were as follows: (1) history of chronic pain other than cLBP; (2) other diseases causing low back pain, such as systemic lupus erythematosus or ankylosing spondylitis; (3) history of head trauma or coma; (4) psychiatric disorders; (5) Beck Depression Inventory (BDI) score > 19 (Beck et al., 1961); (6) contraindication for spinal manipulation, such as spinal surgery, trauma, neoplasm, or inflammation; (7) contraindication for brain MRI scans, such as a cardiac pacer, metal in the orbits, or claustrophobia.

We enrolled age- and sex-matched healthy controls with no history of cLBP (Group 2) from the community through an institution-wide research announcement, advertisements in local newspapers, patient referrals of family and friends, and at health fairs and seminars. The eligibility criteria were as follows: (1) right-handed; (2) 20–70 years of age; (3) no history of cLBP; (4) no pharmacotherapy, physical therapy, or SMT for pain 1 month prior to enrollment; (5) able to understand and sign the written informed consent. The exclusion criteria were the same for Group 1 and Group 2.

This research protocol (No. 2017-520-03-01) was approved by the Institutional Review Board at Shuguang Hospital Affiliated to Shanghai University of Traditional Chinese Medicine, P. R. China. This protocol was registered in the Chinese Clinical Trial Registry (ChiCTR1800015620) and was performed in accordance with the Declaration of Helsinki. Written informed consent was obtained from all participants.

### Procedure

Brain fMRI scans were obtained for patients with cLBP (Group 1) at three time points: before SMT (time point 1, TP1) and within one hour after the first SMT session (time point 2, TP2) and after the sixth SMT session (time point 3, TP3).

Patients in Group 1 were also asked to complete clinical questionnaires prior to the fMRI scan at each time point. Specifically, they were asked to score the severity of their cLBP in terms of VAS (0 = no pain, 10 = strongest imaginable pain) and to complete the C-SFODI forms. Differences in VAS and C-SFODI scores between the three time points were calculated (Table 1).

**Table 1:** Participant information and clinical questionnaire scores.

	**Group 1 (*n* = 14)**	**Group 2 (*n* = 20)**	***F-*value/x^**2**^/Mann Whitney *U-*value**	***P*-value**
Duration of cLBP (months)	44.58 ± 41.66	0 ± 0		
Gender (male/female)	8/6	12/8	1.06	0.30
Age (years)	41.93 ± 12.34	38.50 ± 11.24	0.84	0.41
BDI	2.44 ± 2.73	n/a		
VAS1	50.00 ± 12.86^b,c,d^	0 ± 0^a,b,c^	78.72	<0.001
VAS2	31.43 ± 12.32^a,c,d^	n/a		
VAS3	15.71 ± 10.35^a,b,d^	n/a		
VAS Change 1	−0.37 ± 0.23	n/a		
VAS Change 2	−0.68 ± 0.22	n/a		
VAS Change 3	−0.46 ± 0.38	n/a		
C-SFODI1	27.50 ± 13.57^c,d^	0 ± 0^a,b,c^	31.84	<0.001
C-SFODI2	20.93 ± 9.75^c,d^	n/a		
C-SFODI3	9.00 ± 8.24^a,b,d^	n/a		
C-SFODI Change 1	−0.27 ± 0.39	n/a		
C-SFODI Change 2	−0.71 ± 0.18	n/a		
C-SFODI Change 3	−0.59 ± 0.35	n/a		

a *Significant difference compared to TP1*;

b *significant difference compared to TP2*;

c *significant difference compared to TP3*;

d *significant difference compared to controls (group2). VAS1, VAS2, VAS3, C-SFODI1, C-SFODI2, and C-SFODI3: indicating the VAS and C-SFODI data collected at the same three time points at TP1, TP2, and TP3 as the brain fMRI scans. VAS Change 1 = (VAS2—VAS1)/VAS1; C-SFODI Change 1 = (C-SFODI2—C-SFODI1)/C-SFODI1; VAS Change 2 = (VAS3—VAS1)/VAS1; C-SFODI Change 2 = (C-SFODI3—C-SFODI1)/C-SFODI1; VAS Change 3 = (VAS3—VAS2)/VAS2; C-SFODI Change 3 = (C-SFODI3—C-SFODI2)/C-SFODI2*.

*BDI, Beck depression inventory; C-SFODI, Chinese Short Form Oswestry Disability Index; n/a, not applicable; TP, time point; VAS, visual analog scale*.

Only one brain fMRI scan was obtained for the healthy controls (Group 2), and Group 2 participants did not complete any clinical questionnaires.

### Spinal Manipulative Therapy (SMT)

**Figure 1:**
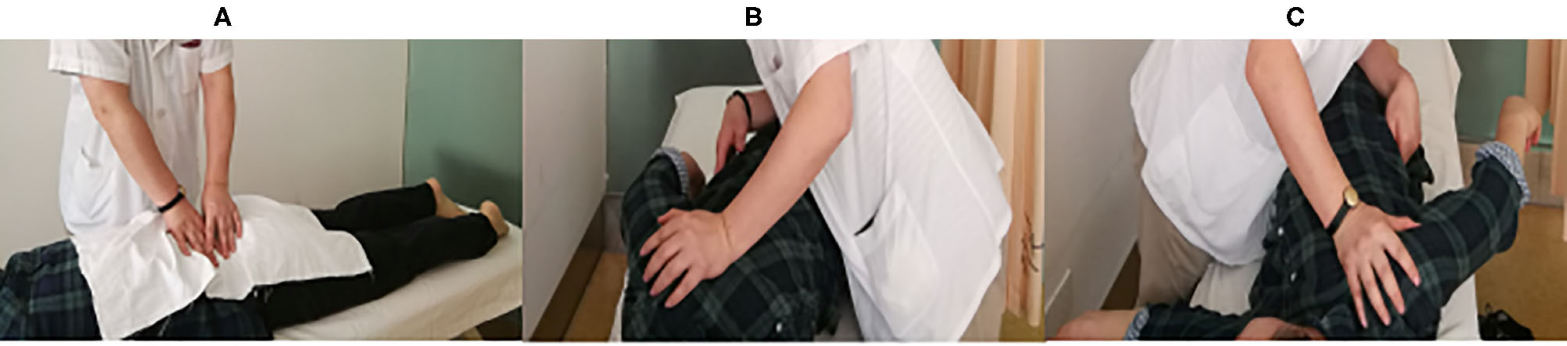
SMT using the Tuina method. **(A)** Massaging and manipulation for lumbar muscular relaxation. **(B,C)** Left and right pulling-rotating manipulations, respectively.

All SMT sessions were performed by the same rehabilitation specialist (JW), who has 28 years of experience. Tuina, a common form of SMT practiced in China, was performed for all patients in this study (Kong et al., 2012; Lee et al., 2017). Tuina combines massage and manipulation, including rolling, kneading, plunking, pushing, and pulling-rotating maneuvers, in the low back area to achieve lumbar muscle relaxation and pain reduction (Figure 1). Each Tuina session lasted about 25 min. All patients in our study underwent a 3-week course of SMT treatment, receiving two Tuina sessions per week for a total of six sessions. There were no complications from the Tuina sessions in our study cohort.

### Brain fMRI Scan

All participants were scanned in the prone position on the same 3T Siemens MR system (Verio, Siemens AG, Erlangen, Germany) with a 12-channel head coil. A sagittal T1-weighted 3D sequence with magnetization prepared rapid gradient echo (MPRAGE) was acquired for structural imaging. An axial T2^*^-weighted echo planner sequence was acquired for functional imaging. The scanning parameters were as follows: (1) T1-weighted MPRAGE in the sagittal plane: TR/TE = 2,050 ms/3.08 ms, flip angle = 9 degrees, field of volume = 220 mm, thickness = 1 mm, resolution = 0.86 × 0.86 mm^2^, and slices = 160; (2) T2^*^ - weighted functional sequence in the axial plane: TR/TE = 2,000 ms/30 ms, flip angle = 90 degrees, field of view = 256 mm × 256 mm, thickness = 4 mm, slice gap = 0.5 mm, matrix = 64 × 64, and slices = 31. A total of 96 time points were acquired. All participants were asked to stay awake and to try not to think of anything during scanning.

**Figure 2:**
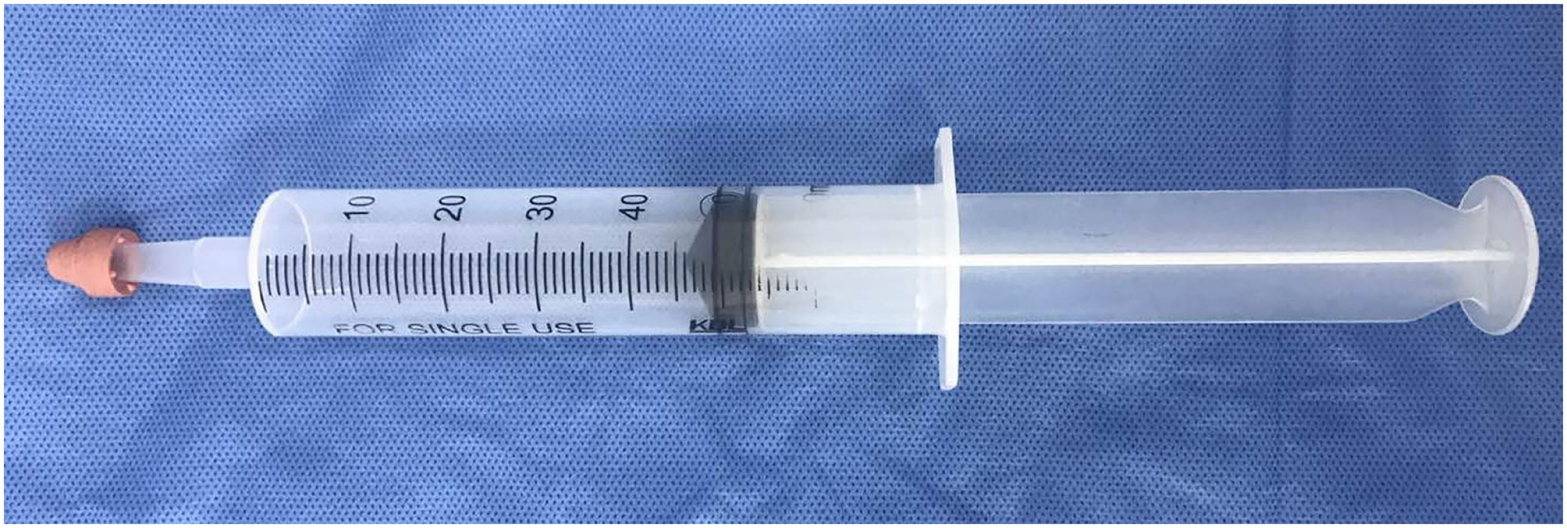
Spot pressure compressor, modified from a 50-ml air-filled syringe. Spot pressure was produced at the rubber tip by manually pressing the syringe.

We used a modified 50-ml air-filled syringe as the MRI-compatible spot pressure compressor for mechanical stimulation (Figure 2). A research assistant inside the MRI scanner applied spot pressure manually during the fMRI scan. The rubber tip of the syringe was applied to the left paraspinal interspace between the fourth and fifth lumbar vertebra, and spot pressure was produced at the rubber tip by manually pressing the syringe, as reported by Kobayashi et al. (2009). Pain related to the spot pressure was recorded using VAS scores. The spot pressure was calibrated such that all participants reached a VAS score of 5.

**Figure 3:**
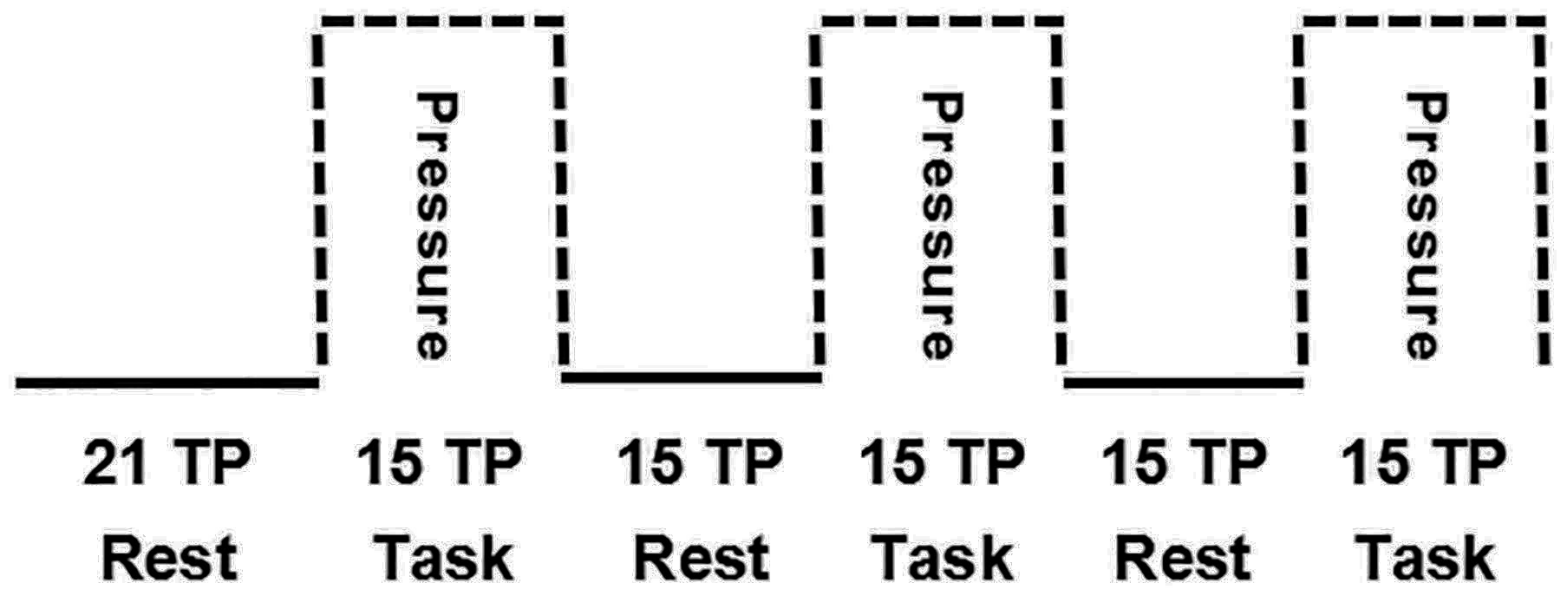
Block design for brain fMRI scans. The first six time points were obtained under resting conditions, followed by six blocks of 15 time points, alternating between blocks taken under “Rest” conditions (without spot pressure) and blocks taken under “Task” conditions (with spot pressure).

The brain fMRI scans were performed in a block design (Figure 3). The first six time points were obtained under resting conditions, followed by six blocks of 15 time points, alternating between blocks taken under “Rest” conditions (without spot pressure) and blocks taken under “Task” conditions (with spot pressure) for a total of 192 s.

### Brain fMRI Processing

Brain fMRI data processing and analysis were carried out using Statistical Parametric Mapping 8 (SPM) (Friston et al., 1994) running on MATLAB (The Mathworks, Inc.). For the time series analysis, the first six time points were discarded to ensure signal stability. The 90 remaining time points were pre-processed using the following procedures: slice-timing correction, realigning, co-registering 3D T1-weighted sequence to the functional image, normalization, re-sampling, and smoothing.

The middle slice was used as the reference for slice-timing correction using the Fourier phase shift interpolation in SPM8. Images from a time series acquired from the same subject were realigned using a least-squares approach and a six-parameter (rigid body) spatial transformation. The first image was used as the reference to which all subsequent scans were realigned, and the values in the volumes were wrapped around the Y direction. For motion susceptibility correction, the criteria for unacceptable images were set to: three translational and rotational head movements of more than or equal to 3.0 mm or 3 degrees, respectively. Sagittal 3D T1-weighted images were co-registered with the mean volume of axial functional images and subsequently segmented using an inbuilt unified segmentation routine in SPM8 (Ashburner and Friston, 2005; Yang et al., 2017; Yoshino et al., 2017). The parameter created by segmentation was then applied to functional images with non-linear normalization to the Montreal Neurological Institute (MNI) brain template, and each voxel was re-sampled to isotropic voxels of 3 × 3 × 3 mm. Gaussian smoothing with a full width at half maximum (FWHM) of 8 mm was used to reduce noise.

After pre-processing, the effect of the task was estimated using a general linear model. Data for each subject was modeled using a boxcar model convolved with the hemodynamic response function. Estimated head movement parameters from realignment were included as covariates at this first level of analysis. Voxel values for task-versus-rest contrast yielded a statistical parametric map from the *t-*test and were then normalized to Z-scores. A corresponding contrast image was created for each patient for group analysis.

There were no participants with head motion > 3 mm of translation or 3 degrees of rotation in our cohort. Translational and rotational head motion did not differ significantly between groups or across time points for Group 1 (Table 2). Therefore, no participants were excluded after imaging analysis.

**Table 2:** Head motion parameters from brain fMRI processing.

**Head motion**	**Group 1 TP1 (mean ± SE)**	**Group 1 TP2 (mean ± SE)**	**Group 1 TP3 (mean ± SE)**	**Group 2 (mean ± SE)**	**x^**2**^-value**	***P*-value**
x	0.05 ± 0.34	0.09 ± 0.44	0.16 ± 0.34	0.25 ± 0.62	4.15	0.25
y	−0.290.47	−0.530.67	−0.370.43	0.840.69	2.00	0.57
z	0.240.46	0.300.36	0.080.44	0.370.48	2.19	0.53
pitch	0.01 ± 0.01	0.01 ± 0.01	0.01 ± 0.01	0.01 ± 0.01	0.71	0.87
roll	0.00 ± 0.00	0.00 ± 0.00	0.00 ± 0.00	0.00 ± 0.00	5.38	0.15
yaw	0.00 ± 0.00	0.00 ± 0.00	0.00 ± 0.01	0.00 ± 0.01	0.71	0.87

*The head motion parameters for the three translational (x, y, and z in mm) and three rotational (pitch, roll, and yaw in degrees) values were obtained from head movement correction during brain fMRI analysis*.

*SE, standard error; TP, time point; fMRI, functional magnetic resonance imaging*.

Group analysis was performed using a fixed-effect model to explore common activation within each group or a time point. A one-sample *t-*test was applied to the contrast images of the subjects in each group or each time point. Finally, we used a random-effect model in performing group analyses to show the difference in the activation area. The paired *t-*test was used to compare the differences in Group 1 at different time points. A two-sample *t-*test was applied to determine areas that showed greater or weaker activation in Group 1 at different time points compared with Group 2. Multiple comparison correction for the results was performed using Monte Carlo simulations using the program AlphaSim by B. D. Ward (http://afni.nimh.nih.gov/pub/dist/doc/manual/AlphaSim.pdf). We estimated the spatial correlation across voxels using the program 3D FWHM. The results for FWHMx, FWHMy, and FWHMz were 8.427, 9.0018, and 7.9957, respectively. According to the AlphSim method, the spatial smoothness was defined by 4 mm, 6 mm, 8 mm, and 10 mm, per the cluster connectivity criteria of rmm = 5. We, therefore, selected a spatial smoothness of 8 mm and a cluster size of 389 voxels. Significant between-group differences in BETA-maps satisfied the criteria of uncorrected *P* < 0.005 at both the voxel level and for cluster sizes of > 26 voxels, corresponding to a corrected *P* < 0.05. Finally, we explored the dissociable anomaly between groups in the whole brain, with statistical significance set at a corrected *P* < 0.05 at the voxel level and for cluster sizes of > 389 voxels.

### Statistical Analysis

We used the statistical software package SSPS 20.0 (IBM SPSS Statistics, Armonk, NY, USA) for statistical analysis. We used a Chi-square test to compare the sex/gender distributions of each group and an independent sample *t-*test to compare the ages of participants in each group.

We performed our initial statistical analysis using the Kruskal-Wallis *H-*test to assess differences in VAS scores, C-SFODI scores, and head motion between Group 1 at TP1, Group 1 at TP2, Group 1 at TP3, and Group 2. The level of statistical significance was set at *P* < 0.05. Once a statistically significant difference was detected, we proceeded with the Mann-Whitney test to determine which pairs of datasets differed significantly: Group 1 at TP1 vs. TP2, Group 1 at TP1 vs. TP3, Group 1 at TP2 vs. TP3, or Group 1 at TP1, TP2, or TP3 vs. Group 2. To avoid type I error, *P* < 0.008 was considered statistically significant, based on a Bonferroni multiple comparison correction (0.05/6 = 0.008).

We also analyzed the correlations of brain activity with the change rates of VAS and C-SFODI scores, respectively. The change rate was defined by the score difference between two time points divided by the score from the initial time point. For example, the change rate for C-SFODI (denoted as C-SFODI Change 2) was obtained via the following: (C-SFODI3—C-SFODI1)/C-SFODI1. The change rate for C-SFODI (denoted as C-SFODI Change 3) was obtained via the following: (C-SFODI3—C-SFODI2)/C-SFODI2. The change rate for VAS (denoted as VAS Change 3) was obtained via the following: (VAS3—VAS2)/VAS2. Variables such as age and head motion were considered in a partial correlation analysis. Bonferroni multiple comparison correction was also used in this partial correlation analysis.

## Results

### Clinical Data

We enrolled 16 patients with cLBP (Group 1). One patient had a metatarsal fracture after the first SMT session and voluntarily withdrew from the study. Another patient with cLBP did not return for the sixth SMT session because his pain subsided. Therefore, a total of 14 patients with cLBP completed all study assessments (mean age ± SD, 41.93 ± 12.34 years). Group 2 included 20 age- and sex/gender-matched healthy controls (mean age ± SD, age 38.50 ± 11.24 years). There were no statistical differences in age (*P* = 0.41) or sex/gender (*P* = 0.30) between the two groups (Table 1).

The VAS scores for Group 1 differed significantly between time points (*P* < 0.001). After one or sixth SMT session (TP2 and TP3), VAS scores were significantly lower than VAS scores before SMT (TP1) (*P* < 0.001 in pair-wise comparisons). C-SFODI scores also differed significantly between time points. Specifically, C-SFODI scores at TP3 differed significantly compared to C-SFODI scores at TP1 and TP2 (*P* ≤ 0.001 in pair-wise comparisons). However, C-SFODI scores did not differ significantly between TP1 and TP2 (*P* = 0.053).

### Brain fMRI Data

At baseline (TP1, before SMT), there were no significant differences in brain activity between Group 1 and Group 2 (*P* > 0.05).

The brain regions with significant activity using a one-sample *t-*test was presented in Table 3. At TP1, there were five brain regions with significant activity: the right cerebellar tonsil, right frontal-temporal lobe, right medial superior frontal gyrus, right supramarginal gyrus, and right frontal lobe. At TP2, there were two significant brain regions, including the right frontal lobe and left inferior temporal gyrus. At TP3, there were six significant brain regions, including the right inferior frontal gyrus, left posterior cingulate, left middle frontal gyrus, left inferior parietal lobe, right inferior parietal lobule, and left supplemental motor area. The peak of the largest cluster of brain activity at a cluster size of 11,237 was in the right frontal lobe at TP2 in Brodmann 10. The peak of the second largest cluster of brain activity at a cluster size of 3,119 was in the right inferior frontal gyrus at TP3 in Brodmann 45. These results indicated that the peak areas of brain responses for the large clusters were located in the right frontal lobe. In Group 2, there were four brain regions with brain activity, including the right inferior frontal gyrus, left superior temporal gyrus, left frontal lobe, and left parietal lobe.

**Table 3:** Summary of brain regions with significant activity.

			**MNI coordinates**				
**Dataset**	**Region**	**Cluster size**	**X**	**Y**	**Z**	**BA**	***t-*value**
Group 1 at TP1	Cerebellar Tonsil_R	958	27	−33	−42	37	4.53
	Frontal-Temporal _R	543	51	12	0	48	5.20
	Frontal_Sup_Medial_R	491	9	66	24	10	5.98
	SupraMarginal_R	398	69	−21	18	22	4.27
	Frontal Lobe_R	1,235	0	−48	72	5	4.70
Group 1 at TP2 (after one session of SMT)	Frontal Lobe_R	11,237	24	69	15	10	8.72
	Inferior Temporal Gyrus_L	582	−57	−45	−18	20	4.49
Group 1 at TP3 (after six sessions of SMT)	Inferior Frontal Gyrus_R	3,119	57	24	18	45	5.50
	Posterior Cingulate_L	1,151	0	−51	21	30	4.63
	Middle Frontal Gyrus_L	685	−51	21	36	44	5.07
	Inferior Parietal Lobule_L	732	−48	−60	48	40	3.98
	Inferior Parietal Lobule_R	463	63	−30	30	40	3.13
	Supp_Motor_Area_L	717	0	−3	72	6	4.64
Group 2	Inferior Frontal Gyrus_R	365	57	18	−6	38	5.85
	Superior Temporal Gyrus_L	327	−57	9	−3	22	4.50
	Frontal Lobe_L	339	−33	57	27	46	3.60
	Parietal Lobe_L	263	−57	−54	48	40	4.06

*Brain regions with significant activity from one-sample t-test were identified with P <0.005 and cluster size > 26 voxels (activity volume)*.

*BA, Brodmann area; L, left; MNI, Montreal Neurological Institute; R, right; Sup, superior; Group 1, patients with cLBP; Group 2, healthy controls; TP, time point*.

There were also no significant within-group longitudinal differences for Group 1 (*P* > 0.05 for all pair-wise comparisons). No longitudinal comparisons were assessed for Group 2, as only one brain fMRI scan was obtained for healthy controls.

**Figure 4:**
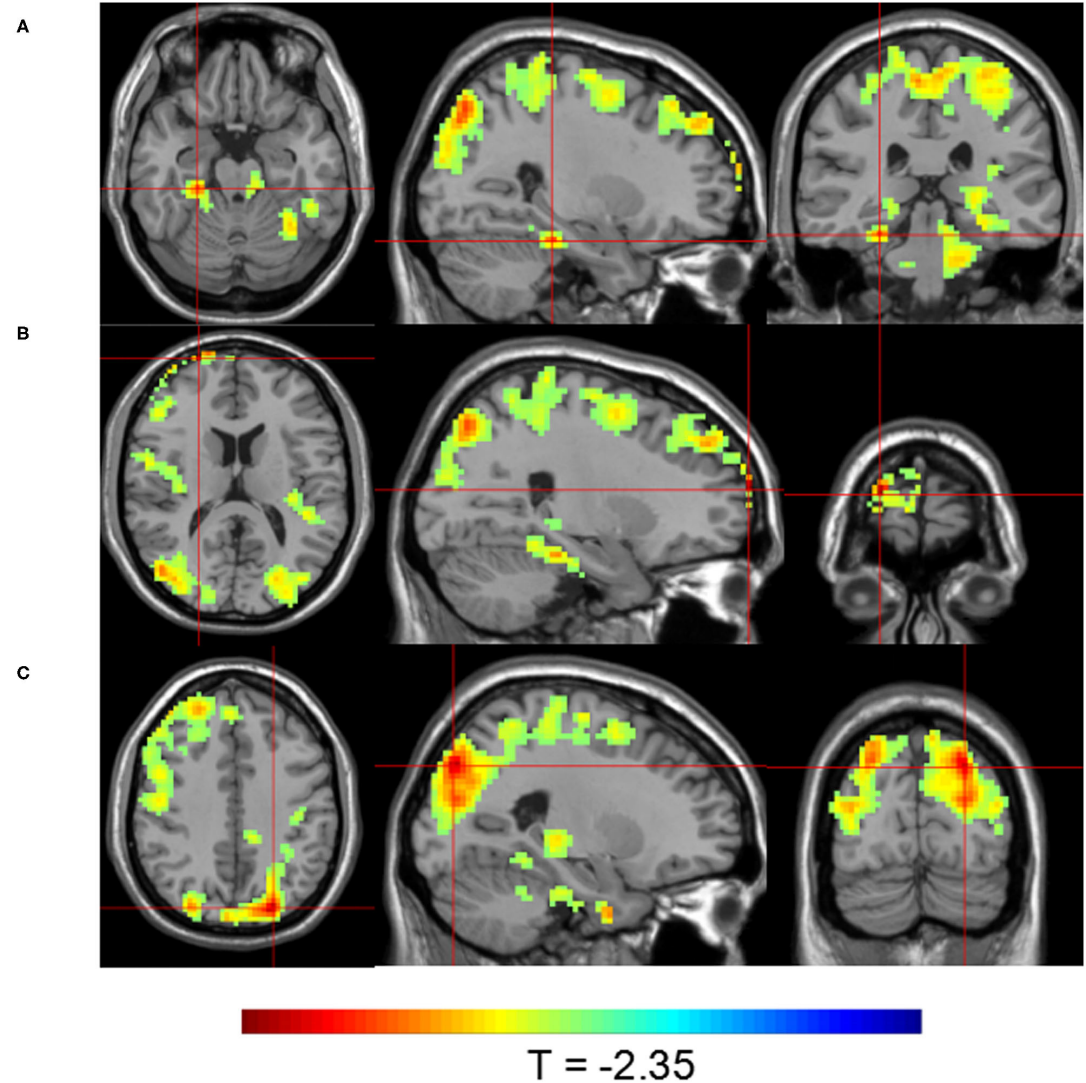
Brain activity maps showing greater brain activity in Group 1 at TP2 (after one SMT session) compared to Group 2. Greater activity in **(A)** the right parahippocampal gyrus; **(B)** the right dorsolateral prefrontal cortex; and **(C)** the left precuneus.

Group 1 at TP2 (after the first SMT session) exhibited significantly higher activity than Group 2 in the right parahippocampal gyrus, right dorsolateral prefrontal cortex, and left precuneus (*P* < 0.05; Table 4, Figure 4). The peak of the largest cluster of brain activity, with a cluster size of 1,559, was in the right dorsolateral prefrontal cortex in Brodmann 9. Subcortical structures, such as the thalamus, also showed greater activity in Group 1 at TP2, with a cluster size of 164.

**Table 4:** Summary of significant differences in brain activity between Group 1 and Group 2.

**Comparison**	**Region**	**Cluster size**	**MNI coordinates**			**BA**	***t-*value**
			**X**	**Y**	**Z**		
Group 1 at TP2 vs. Group 2	Parahippocampal gyrus_R	957	27	−30	−21	36	4.62
	Pons	124					
	Middle Temporal Gyrus_R	96					
	Superior Temporal Gyrus_R	90					
	Middle Temporal Pole_R	77					
	Temporal Pole_R	58				38	
	Dorsolateral Prefrontal Cortex_R	1,559	24	69	15	9	6.34
	Middle Frontal Gyrus_R	585					
	Inferior Frontal Gyrus_R	144					
	Middle Frontal Gyrus_R	105					
	Inferior Frontal Orbital Gyrus_R	72					
	Precuneus_L	1,471	−24	−81	39	19	5.72
	Cuneus_L	158					
	Paracentral Lobule_L	157					
	Postcentral Gyrus_L	87					
Group 1 at TP3 vs. Group 2	Posterior Cingulate	640	9	−54	3	18	3.92
	Thalamus	164					
	Left Brainstem	89					
	Right Brainstem	87					
	Inferior Frontal Gyrus_R	450	57	39	−6	45	3.98
	Middle Frontal Gyrus_R	230					
	Inferior Frontal Triangle Gyrus_R	173					

*Brain regions were identified with P <0.05 and cluster size > 389*.

*BA, Brodmann area; L, left; R, right; MNI, Montreal Neurological Institute; Group 1, patients with cLBP; Group 2, healthy controls; TP, time point*.

**Figure 5:**
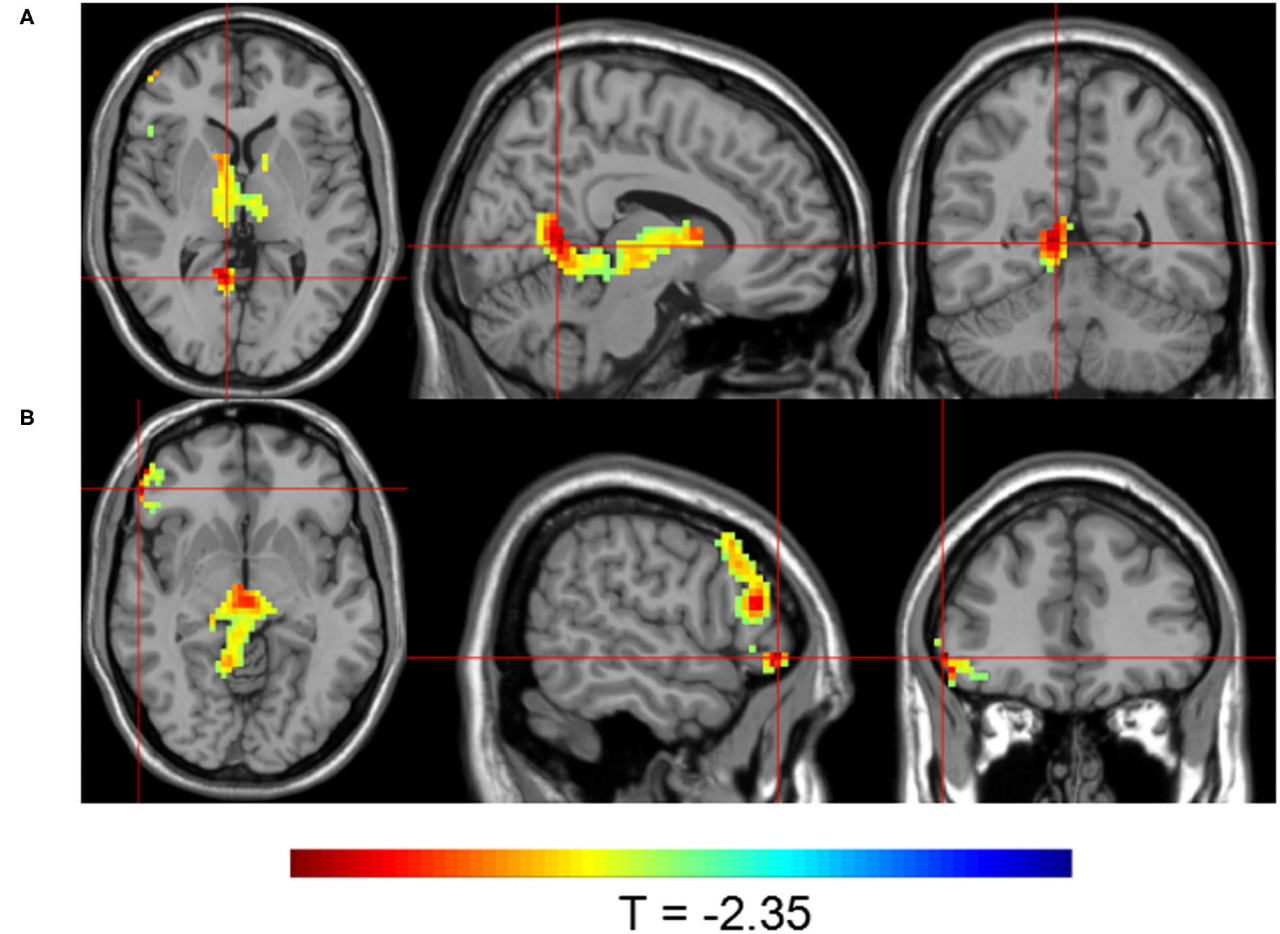
Brain activity maps showing greater brain activity in Group 1 at TP3 (after six SMT sessions) compared to Group 2. Greater activity in **(A)** the posterior cingulate and **(B)** the right inferior frontal gyrus.

Group 1 at TP3 (after the sixth SMT session) exhibited significantly higher activity than Group 2 in the posterior cingulate gyrus and right inferior frontal gyrus (*P* < 0.05; Table 4, Figure 5). The peak of the larger cluster of brain activity, with a cluster size of 640, was in the posterior cingulate in Brodmann 18.

**Figure 6:**
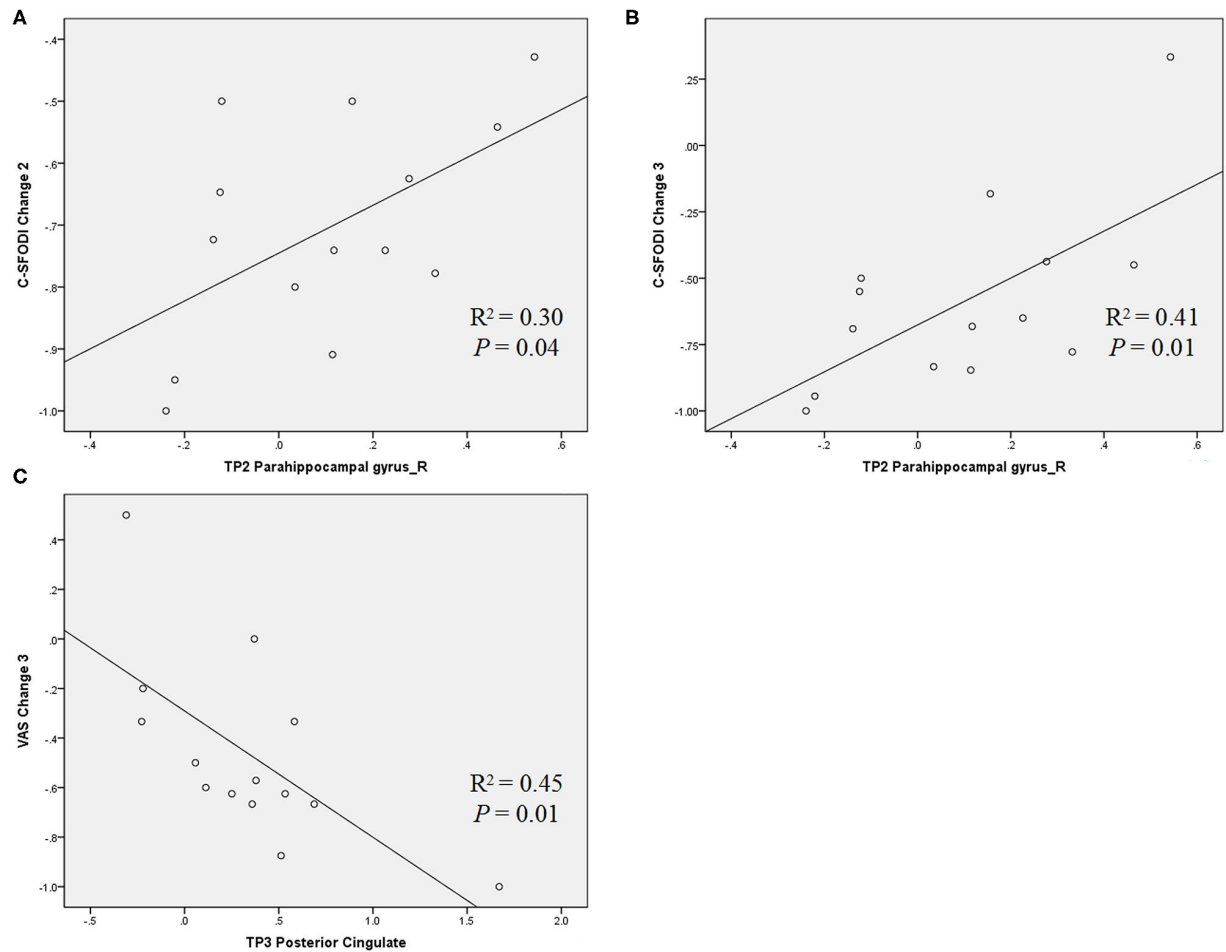
Scatter diagrams showing the correlations between brain activity and change rates of VAS and C-SFODI scores, respectively. **(A)** Positive correlation between brain activity in the right parahippocampal at TP2 and the change rate of C-SFODI between TP1 and TP3 [(C-SFODI3—C-SFODI1)/C-SFODI1]; **(B)** positive correlation between brain activity in the right parahippocampal gyrus at TP2 and the change rate of C-SFODI between TP2 and TP3 [(C-SFODI3—C-SFODI2)/C-SFODI2]; **(C)** negative correlation between brain activity in the posterior cingulate at TP3 and the change rate of VAS between TP2 and TP3 [(VAS3—VAS2)/VAS2].

Significant correlations of brain activity with the change rates of VAS and C-SFODI scores for Group 1 were presented in Table 5. Brain activity in the right parahippocampal gyrus at TP2 correlated positively with the change rates of C-SFODI scores between TP1 and TP3 and between TP2 and TP3 (*P* = 0.04 and 0.01, respectively). Brain activity in the posterior cingulate at TP3 correlated negatively with the change rate of VAS scores between TP2 and TP3 (*P* = 0.01; Figure 6). After correcting for age and head motion, brain activity in the posterior cingulate at TP3 correlated negatively with VAS scores at TP3 (*P* = 0.03), with a correlation coefficient of −0.793. We found no significant differences in this partial correlation analysis after Bonferroni correction, performed for ten comparisons with a corrected significance level of *P* < 0.005.

**Table 5:** Correlations between brain activity and clinical scores after SMT.

	**Brain area**	**Clinical features**	**Correlation coefficient**	***P*-value**
Group 1 at TP2 (after one SMT session)	Parahippocampal gyrus_R	C-SFODI Change 2	0.54	0.04
		C-SFODI Change 3	0.66	0.01
Group 1 at TP3 (after six SMT sessions)	Posterior Cingulate	VAS Change 3	−0.64	0.01

*C-SFODI Change 2 = (C-SFODI3—C-SFODI1)/C-SFODI1; VAS Change 3 = (VAS3—VAS2)/VAS2; C-SFODI Change 3 = (C-SFODI3—C-SFODI2)/C-SFODI2*.

*TP2, time point 2; TP3, time point 3; R, right; VAS, Visual analog scale; C-SFODI, Chinese Short Form Oswestry Disability Index*.

## Discussion

Our study detected greater brain activity in several brain regions, including the right parahippocampal gyrus, left precuneus, and posterior cingulate, in patients with cLBP after SMT compared to matched healthy controls. These brain regions are part of the default mode network (DMN), suggesting that activity in the DMN may be a neural correlate of cLBP and DMN alterations may reflect responses to pain management with SMT in patients with cLBP.

We used real-time spot pressure as a mechanical stimulus in our study. This method has been used in prior research to mimic the pain that patients experience when sitting, walking, or standing (Mansour et al., 2018). A report by Ng et al. (2018) showed significantly different brain activity between patients with cLBP and healthy controls during pressure stimulation in the low back area. Kobayashi et al. (2009) identified a matrix of brain regions with alterations in activity in response to the pressure stimuli in the low back area. Specifically, they reported that patients with cLBP had increased activation in the right insula, posterior cingulate cortex and supplementary motor area than the healthy volunteers during pressure stimulation (Kobayashi et al., 2009). Our results were generally in line with their report, as we also identified brain regions with significantly increased activity, including regions of the frontal lobe, when spot pressure was applied at baseline (prior to SMT). However, we did not observe any statistically significant differences in brain activity when comparing patients with cLBP before SMT to healthy controls. We speculate that the disparities between our findings and theirs may be due to our more stringent approach for data analysis, having employed corrections for multiple comparisons. Nevertheless, our findings were consistent with a prior study by Giesecke et al. (2004) that used thumbnail-generated pressure stimuli for eliciting equally painful responses in all participants, similar to our focal pressure approach, and they found no significant differences in neuronal activations among the patients with idiopathic cLBP, the patients with fibromyalgia and healthy controls.

It should be noted that our method for the real-time application of spot pressure was similar but also had specific differences compared to previously published studies. For instance, Matsuo et al. (2017) used a fixed pressure of 500 kPa to stimulate pain in the low back area. They reported deactivation of the dorsolateral prefrontal cortex and anterior cingulate cortex in patients with cLBP compared to healthy controls. In our study, we varied the intensity of the spot pressure applied, rather than using a fixed pressure for all participants, to achieve the same subjective feeling of pain (VAS score = 5) in the low back area of all participants. Our rationale was that the same objective magnitude of mechanical pressure might produce different pain intensity among participants, which would add an unnecessary confounding variable to our study.

Alterations to the DMN have previously been reported in patients with cLBP. The DMN consists of three major subdivisions: the ventral portion of the medial prefrontal cortex, the dorsal portion of the medial prefrontal cortex, and the posterior cingulate cortex and adjacent precuneus. This network functions to regulate emotions and retrieve memories (Ward et al., 2014; Raichle, 2015). A study by Baliki et al. (2008) showed abnormal activity in the medial part of the prefrontal cortex in patients with cLBP. Zhang et al. (2019) observed altered intrinsic brain activity parameters in patients with cLBP compared to healthy controls. Their study showed that the patients with cLBP had increased amplitude of low-frequency fluctuation (ALFF) values in the hippocampal/parahippocampal gyrus but decreased ALFF values in the remaining DMN regions when their spontaneous low back pain intensity increased after the pain-exacerbating maneuver (Zhang et al., 2019). Zhou et al. (2018) found lower ALFF values in DMN regions in patients with discogenic low back pain and leg pain compared with healthy controls. In our study, patients with cLBP showed greater activity compared to healthy controls in the right parahippocampal gyrus and left precuneus after the first SMT session and in the posterior cingulate after the sixth SMT session. These brain regions are part of or are closely connected to regions of the DMN. We also observed positive correlations of brain activity in these regions with VAS and C-SFODI scores. Therefore, our results support the notion that activity in the DMN is a neural correlate of cLBP, and the DMN may be involved in the analgesic effect of SMT and the brain responses to pain management in patients with cLBP after SMT.

Our results are consistent with findings from prior studies on therapy-induced alterations in brain activity in patients with cLBP. One study by Seminowicz et al. (2011) reported a reversal of brain activity in the left dorsolateral prefrontal cortex to normal values after spine surgery and facet joint injections. Another study by Ceko et al. (2015) reported that connectivity between the insula, dorsolateral prefrontal lobe, and cognitive networks are partly restored in patients with cLBP after pharmacological treatment. Ellingsen et al. (2018) used brain fMRI method to assess brain responses underpinning pain anticipation and fear of movement in patients with cLBP and treatment responses following SMT. In their study, brain fMRI scans were obtained while the participants were watching video clips showing back-straining or neutral physical exercises in the scanner. Patients with cLBP, compared to the healthy controls, showed higher blood oxygen level–dependent signal in brain circuitry implicated in salience, social cognition, and mentalizing while watching the video. However, involvement of the brain circuitry was decreased after subsequent SMT, indicating SMT-induced reduction in anticipated pain and fear (Ellingsen et al., 2018). However, their study focused on the psychological factors of patients with cLBP, whereas our study focused on the physiological responses of the brain to pain after SMT treatment. Nevertheless, our study was generally in agreement with the results of others regarding altered brain activity in patients with cLBP undergoing pain management. We observed greater activity in the dorsolateral prefrontal cortex in the patients with cLBP after the first SMT session compared to the healthy controls, indicating an SMT-associated effect on brain activity.

Our study detected greater activity in the thalamus in patients with cLBP after SMT compared to healthy controls, indicating that subcortical structures may also be involved in the response of the brain to pain. The involvement of the thalamus in cLBP has been reported previously. Gussew et al. (2011) examined the metabolic changes in patients with cLBP and reported decreased myo-inositol and N-acetyl-aspartate levels in the thalamus, indicating glial cell, and neuronal loss. Another study by Didehdar et al. (2020) reported increased N-acetyl aspartate and choline in the thalamus and dorsolateral prefrontal cortex after SMT compared to sham treatment. Taken together, prior reports and the current study provide evidence supporting the involvement of subcortical structures, such as the thalamus, in pain relief after SMT through alterations in brain metabolism and activity.

Our study identified correlations between brain activity and questionnaire scores for pain and quality of life. These findings were encouraging as they associated fMRI brain activity findings, which are relatively new in this field, with established measures that have been routinely used in clinical practice for assessing the well-being of patients with cLBP. Our study showed that higher brain activity in the right parahippocampal gyrus after one SMT session and lower brain activity in the posterior cingulate after six SMT sessions were correlated with reduced pain and improved quality of life. Our results demonstrate the beneficial effects of SMT on both pain relief and function.

It should be noted that a well-designed longitudinal study should include comparisons of different time points in the patients with cLBP after SMT to the matched longitudinal time point assessments for the healthy control group. A single study assessment for the healthy controls as in our study was not adequate, which would lead to challenges for reaching a meaningful conclusion. In particular, a longitudinal brain fMRI research should be designed in a stringent method as brain activity could have potentially fluctuated in the patients and controls even within a short period of time. In addition, a sham condition such as a “sham” spinal manipulation to mimic a true SMT would have helped to minimize bias through randomization. With the patients having no knowledge of treatment assignment, i.e., a true SMT vs. a “sham” SMT, we would have been able to control for variables such as the effect of patients’ expectation from the treatment and the potential placebo effect on the brain activity. Nevertheless, our study showed no significant differences in brain activity at baseline between the patients with cLBP prior to SMT and the healthy controls but significant differences were noted when comparing the patients after SMT to the healthy controls. Therefore, it was reasonable to consider that SMT altered the brain activity in the patients with cLBP, although these changes were too slight to show the significant differences in the patients with cLBP at different time points.

There were several limitations to this study. First, our sample size was small and heterogeneous, thus limiting our ability to identify subtle changes in brain activity. For example, we did not observe statistically significant changes in brain activity over time within the group of patients with cLBP after SMT, which was unexpected. We speculate that the lack of significant longitudinal within-group changes might be partly due to the small sample size. It may also be partly due to our stringent data analysis approach with corrections for multiple comparisons. Second, our study was limited by the wide range of ages of the participants, which may have generated greater variability than expected in their brain activity maps, and our small cohort was not powered to assess the effects of age on brain activity. We did not consider age as a covariate in our analysis because the patient group and the healthy control group were matched by age. Nevertheless, we understand the prudence of focusing our pain research on a specific age group, especially older adults, because back pain is much more prevalent in that population, significantly affecting their independent living and their quality of life. This pilot study has provided critical preliminary data for estimating the effect sizes of pain management on brain structure and function in different age groups, which we will use to plan larger trials in the future. Third, we did not acquire follow-up data on the patients with cLBP treated with SMT. Therefore, we could not assess the long-term trajectory of alterations in brain activity. It is therefore unknown if the brain changes were transient with a reversal to the pre-treatment, persistent, or partially recovered. In addition, we did not have data on how long the pain relief lasted after SMT. In the future, an additional follow-up assessment, such as a 1-year interval assessment, should be obtained to evaluate changes in the brain responses identified in this study. Fourth, patients in our study were recruited from outpatient pain clinics as walk-in patients, and their low back pain was usually less severe compared to that of patients in an inpatient setting who may have more debilitating pain. Therefore, caution should be exercised when generalizing our study results to patients with very severe back pain leading to hospitalization and invasive treatment, such as spinal surgery and pain injections. Lastly, we only obtained one brain fMRI scan for each of our healthy controls during the entire study, assuming that their brain activity would not change significantly within the 3-week period. This approach was not optimal, as brain fMRI scans should have been performed at matched time points for the patients and healthy controls. In addition, our study was further limited for lack of sham conditions such as a “sham” spinal manipulation to mimic a true SMT. Brain activity for pain response may be affected by various factors such as the anticipation of the treatment, the true treatment effect, the experience of going through the treatment session either a true SMT or a “sham” SMT, etc. Randomization of treatment assignment groups would have helped to assess the effect of SMT on brain activity in patients with cLBP. Nevertheless, our study had some merit in that we were able to obtain pilot data for hypothesis generating for a planned multicenter trial assessing brain activity in patients with cLBP undergoing SMT. Our study should motivate further research to understand how the brain responds to cLBP and how brain responsesmay be altered by treatment to provide relief from pain.

In conclusion, our study identified significant alterations in brain activity in the DMN regions of patients with cLBP after SMT treatment. In addition, our data support activity in the DMN as an underlying neural correlate of cLBP, and alterations to the DMN may be a potential neuroimaging biomarker for assessing brain responses to back pain management.

## Data Availability Statement

The datasets generated for this study are available on request to the corresponding author.

## Ethics Statement

The studies involving human participants were reviewed and approved by the institutional review board of Shuguang Hospital Affiliated with Shanghai University of Traditional Chinese Medicine. The patients/participants provided their written informed consent to participate in this study. Written informed consent was obtained from the individual(s) for the publication of any potentially identifiable images or data included in this article.

## Author Contributions

WT, SZ, and JW conceived and designed the study. WT, YY, CZ, ZC, and BC drafted and revised the manuscript. All authors participated in the collection and analysis of the data.

## Conflict of Interest

The authors declare that the research was conducted in the absence of any commercial or financial relationships that could be construed as a potential conflict of interest.

## Abbreviations

ALFF: amplitude of low-frequency fluctuations
BDI: Beck depression inventory
cLBP: chronic low back pain
C-SFODI: Chinese Short Form Oswestry Disability Index questionnaire
DMN: default mode network
fMRI: functional magnetic resonance imaging
MR: magnetic resonance
SMT: spinal manipulative therapy
TP: time point
VAS: visual analog scales.

## References

[B1] Ashburner J. Friston K. J. (2005). Unified segmentation. Neuroimage 26, 839–851. 10.1016/j.neuroimage.2005.02.018 15955494

[B2] Baliki M. N. Geha P. Y. Jabakhanji R. Harden N. Schnitzer T. J. Apkarian A. V. (2008). A preliminary fMRI study of analgesic treatment in chronic back pain and knee osteoarthritis. Mol. Pain 4:47. 10.1186/1744-8069-4-47 18950528 PMC2584040

[B3] Beck A. T. Ward C. H. Mendelson M. Mock J. Erbaugh J. (1961). An inventory for measuring depression. Arch. Gen. Psychiatry 4, 561–571. 10.1001/archpsyc.1961.01710120031004 13688369

[B4] Bervoets D. C. Luijsterburg P. A. Alessie J. J. Buijs M. J. Verhagen A. P. (2015). Massage therapy has short-term benefits for people with common musculoskeletal disorders compared to no treatment: a systematic review. J. Physiother. 61, 106–116. 10.1016/j.jphys.2015.05.018 26093806

[B5] Bronfort G. Haas M. Evans R. Leininger B. Triano J. (2010). Effectiveness of manual therapies: the UK evidence report. Chiropr. Osteopat 18:3. 10.1186/1746-1340-18-3 20184717 PMC2841070

[B6] Burton A. K. Balague F. Cardon G. Eriksen H. R. Henrotin Y. Lahad A.. (2006). Chapter 2. European guidelines for prevention in low back pain: November 2004. Eur. Spine J. 15(Suppl. 2), S136–S168. 10.1007/s00586-006-1070-3 16550446 PMC3454541

[B7] Ceko M. Shir Y. Ouellet J. A. Ware M. A. Stone L. S. Seminowicz D. A. (2015). Partial recovery of abnormal insula and dorsolateral prefrontal connectivity to cognitive networks in chronic low back pain after treatment. Hum. Brain Mapp. 36, 2075–2092. 10.1002/hbm.22757 25648842 PMC6869701

[B8] Didehdar D. Kamali F. Yoosefinejad A. K. Lotfi M. (2020). The effect of spinal manipulation on brain neurometabolites in chronic nonspecific low back pain patients: a randomized clinical trial. Ir. J. Med. Sci. 189, 543–550. 10.1007/s11845-019-02140-2 31773541

[B9] Ellingsen D. M. Napadow V. Protsenko E. Mawla I. Kowalski M. H. Swensen D.. (2018). Brain mechanisms of anticipated painful movements and their modulation by manual therapy in chronic low back pain. J. Pain 19, 1352–1365. 10.1016/j.jpain.2018.05.012 30392530 PMC6220681

[B10] Friston K. J. Worsley K. J. Frackowiak R. S. Mazziotta J. C. Evans A. C. (1994). Assessing the significance of focal activations using their spatial extent. Hum. Brain Mapp. 1, 210–220. 10.1002/hbm.460010306 24578041

[B11] Giesecke T. Gracely R. H. Grant M. A. Nachemson A. Petzke F. Williams D. A.. (2004). Evidence of augmented central pain processing in idiopathic chronic low back pain. Arthritis Rheum. 50, 613–623. 10.1002/art.20063 14872506

[B12] Gussew A. Rzanny R. Gullmar D. Scholle H. C. Reichenbach J. R. (2011). 1H-MR spectroscopic detection of metabolic changes in pain processing brain regions in the presence of non-specific chronic low back pain. Neuroimage 54, 1315–1323. 10.1016/j.neuroimage.2010.09.039 20869447

[B13] Kobayashi Y. Kurata J. Sekiguchi M. Kokubun M. Akaishizawa T. Chiba Y.. (2009). Augmented cerebral activation by lumbar mechanical stimulus in chronic low back pain patients: an FMRI study. Spine 34, 2431–2436. 10.1097/BRS.0b013e3181b1fb76 19789470

[B14] Kong L. J. Fang M. Zhan H. S. Yuan W. A. Pu J. H. Cheng Y. W.. (2012). Tuina-focused integrative chinese medical therapies for inpatients with low back pain: a systematic review and meta-analysis. Evid. Based Complement. Alternat. Med. 2012:578305. 10.1155/2012/578305 23346207 PMC3543824

[B15] Konno S. I. Sekiguchi M. (2018). Association between brain and low back pain. J. Orthop. Sci. 23, 3–7. 10.1016/j.jos.2017.11.007 29167069

[B16] Kregel J. Meeus M. Malfliet A. Dolphens M. Danneels L. Nijs J.. (2015). Structural and functional brain abnormalities in chronic low back pain: a systematic review. Semin. Arthritis Rheum. 45, 229–237. 10.1016/j.semarthrit.2015.05.002 26092329

[B17] Lee N. W. Kim G. H. Heo I. Kim K. W. Ha I. H. Lee J. H.. (2017). Chuna (or Tuina) manual therapy for musculoskeletal disorders: a systematic review and meta-analysis of randomized controlled trials. Evid. Based Complement. Alternat. Med. 2017:8218139. 10.1155/2017/8218139 29441114 PMC5758860

[B18] Manchikanti L. Singh V. Datta S. Cohen S. P. Hirsch J. A. P. (2009). American society of interventional pain, comprehensive review of epidemiology, scope, and impact of spinal pain. Pain Phys. 12, E35–E70. 19668291

[B19] Mansour Z. M. Martin L. E. Lepping R. J. Kanaan S. F. Brooks W. M. Yeh H. W.. (2018). Brain response to non-painful mechanical stimulus to lumbar spine. Brain Sci. 8:41. 10.3390/brainsci8030041 29494490 PMC5870359

[B20] Matsuo Y. Kurata J. Sekiguchi M. Yoshida K. Nikaido T. Konno S. I. (2017). Attenuation of cortical activity triggering descending pain inhibition in chronic low back pain patients: a functional magnetic resonance imaging study. J. Anesth. 31, 523–530. 10.1007/s00540-017-2343-1 28365848

[B21] Meier M. L. Hotz-Boendermaker S. Boendermaker B. Luechinger R. Humphreys B. K. (2014). Neural responses of posterior to anterior movement on lumbar vertebrae: a functional magnetic resonance imaging study. J. Manipulative Physiol. Ther. 37, 32–41. 10.1016/j.jmpt.2013.09.004 24229849

[B22] Meucci R. D. Fassa A. G. Faria N. M. (2015). Prevalence of chronic low back pain: systematic review. Rev. Saude Publica 49:1. 10.1590/S0034-8910.2015049005874 26487293 PMC4603263

[B23] Ng S. K. Urquhart D. M. Fitzgerald P. B. Cicuttini F. M. Hussain S. M. Fitzgibbon B. M. (2018). The relationship between structural and functional brain changes and altered emotion and cognition in chronic low back pain brain changes: a systematic review of MRI and fMRI studies. Clin. J. Pain 34, 237–261. 10.1097/AJP.0000000000000534 28719509

[B24] Raichle M. E. (2015). The brain's default mode network. Annu. Rev. Neurosci. 38, 433–447. 10.1146/annurev-neuro-071013-014030 25938726

[B25] Rainville J. Nguyen R. Suri P. (2009). Effective conservative treatment for chronic low back pain. Semin. Spine Surg. 21, 257–263. 10.1053/j.semss.2009.08.009 20161564 PMC2805825

[B26] Ruddock J. K. Sallis H. Ness A. Perry R. E. (2016). Spinal manipulation vs sham manipulation for nonspecific low back pain: a systematic review and meta-analysis. J. Chiropr. Med. 15, 165–183. 10.1016/j.jcm.2016.04.014 27660593 PMC5021904

[B27] Seminowicz D. A. Wideman T. H. Naso L. Hatami-Khoroushahi Z. Fallatah S. Ware M. A.. (2011). Effective treatment of chronic low back pain in humans reverses abnormal brain anatomy and function. J. Neurosci. 31, 7540–7550. 10.1523/JNEUROSCI.5280-10.2011 21593339 PMC6622603

[B28] Sharma H. A. Gupta R. Olivero W. (2011). fMRI in patients with lumbar disc disease: a paradigm to study patients over time. J. Pain Res. 4, 401–405. 10.2147/JPR.S24393 22247623 PMC3255994

[B29] Shmagel A. Foley R. Ibrahim H. (2016). Epidemiology of chronic low back pain in US adults: data from the 2009-2010 National health and nutrition examination survey. Arthritis Care Res. 68, 1688–1694. 10.1002/acr.22890 26991822 PMC5027174

[B30] Ward A. M. Schultz A. P. Huijbers W. van Dijk K. R. Hedden T. Sperling R. A. (2014). The parahippocampal gyrus links the default-mode cortical network with the medial temporal lobe memory system. Hum. Brain Mapp. 35, 1061–1073. 10.1002/hbm.22234 23404748 PMC3773261

[B31] Xu M. Yan S. Yin X. Li X. Gao S. Han R.. (2013). Acupuncture for chronic low back pain in long-term follow-up: a meta-analysis of 13 randomized controlled trials. Am. J. Chin. Med. 41, 1–19. 10.1142/S0192415X13500018 23336503

[B32] Yang Q. Wang Z. Yang L. Xu Y. Chen L. M. (2017). Cortical thickness and functional connectivity abnormality in chronic headache and low back pain patients. Hum. Brain Mapp. 38, 1815–1832. 10.1002/hbm.23484 28052444 PMC6867133

[B33] Yoshino A. Okamoto Y. Doi M. Otsuru N. Okada G. Takamura M.. (2017). Regional brain functions in the resting state indicative of potential differences between depression and chronic pain. Sci Rep. 7:3003. 10.1038/s41598-017-03522-1 28592893 PMC5462802

[B34] Yu R. Gollub R. L. Spaeth R. Napadow V. Wasan A. Kong J. (2014). Disrupted functional connectivity of the periaqueductal gray in chronic low back pain. Neuroimage Clin. 6, 100–108. 10.1016/j.nicl.2014.08.019 25379421 PMC4215524

[B35] Yuan W. A. Shen Z. B. Xue L. Tan W. L. Cheng Y. W. Zhan S. H.. (2015). [Effect of spinal manipulation on brain functional activity in patients with lumbar disc herniation]. Zhejiang Da Xue Xue Bao Yi Xue Ban 44, 124–30, 137. 26038129 10.3785/j.issn.1008-9292.2015.03.002 PMC10396920

[B36] Zhang B. Jung M. Tu Y. Gollub R. Lang C. Ortiz A.. (2019). Identifying brain regions associated with the neuropathology of chronic low back pain: a resting-state amplitude of low-frequency fluctuation study. Br. J. Anaesth. 123, e303–e311. 10.1016/j.bja.2019.02.021 30948036 PMC6676015

[B37] Zheng G. Zhao X. Liu G. Zhang L. (2002). Reliability of the modified oswestry disability index for evaluating patients with low back pain. Chin. J Spine Spinal Cord 12, 13–15.

[B38] Zhou F. Gu L. Hong S. Liu J. Jiang J. Huang M.. (2018). Altered low-frequency oscillation amplitude of resting state-fMRI in patients with discogenic low-back and leg pain. J. Pain Res. 11, 165–176. 10.2147/JPR.S151562 29386913 PMC5767087

